# Bioindustrial manufacturing readiness levels (BioMRLs) as a shared framework for measuring and communicating the maturity of bioproduct manufacturing processes

**DOI:** 10.1093/jimb/kuac022

**Published:** 2022-09-23

**Authors:** Michael J Smanski, Aristos Aristidou, Ryan Carruth, John Erickson, Mark Gordon, Sandeep B Kedia, Kelvin H Lee, Darcy Prather, John E Schiel, Heather Schultheisz, Thomas P Treynor, Steven L Evans, Douglas C Friedman, Melanie Tomczak

**Affiliations:** BioMADE, Saint Paul, MN 55108, USA; Department of Biochemistry, Molecular Biology, and Biophysics, University of Minnesota, Saint Paul, MN 55108, USA; Cargill, Incorporated, Minneapolis, MN 55391, USA; Superbrewed Foods, Incorporated, New Castle, DE 19720, USA; National Institute of Innovation in Manufacturing Biopharmaceuticals, Newark, DE 19713, USA; Mfg Strategy, Inc., Tampa, FL 33613, USA; National Institute of Innovation in Manufacturing Biopharmaceuticals, Newark, DE 19713, USA; National Institute of Innovation in Manufacturing Biopharmaceuticals, Newark, DE 19713, USA; Kalion, Incorporated, Milton, MA 02186, USA; National Institute of Standards and Technology, Gaithersburg, MD 20899, USA; Genomatica, Inc., San Diego, CA 92121,USA; R2DIO Inc., Berkeley, CA 94702–1102, USA; BioMADE, Saint Paul, MN 55108, USA; BioMADE, Saint Paul, MN 55108, USA; BioMADE, Saint Paul, MN 55108, USA

**Keywords:** BioMRL, Bioproduct, Bioeconomy, Bioindustrial manufacturing

## Abstract

Readiness level (RL) frameworks such as technology readiness levels and manufacturing readiness levels describe the status of a technology/manufacturing process on its journey from initial conception to commercial deployment. More importantly, they provide a roadmap to guide technology development and scale-up from a ‘‘totality of system’’ approach. Commercialization risks associated with too narrowly focused R&D efforts are mitigated. RLs are defined abstractly so that they can apply to diverse industries and technology sectors. However, differences between technology sectors make necessary the definition of sector specific RL frameworks. Here, we describe bioindustrial manufacturing readiness levels (BioMRLs), a classification system specific to bioindustrial manufacturing. BioMRLs will give program managers, investors, scientists, and engineers a shared vocabulary for prioritizing goals and assessing risks in the development and commercialization of a bioindustrial manufacturing process.

## Growing Importance of the Bioindustrial Economy

Bioindustrial manufacturing describes the use of live organisms or active biomolecules to produce goods at scale. A major category, microbial fermentation, is already widely used in industry for food and beverage production (Hugenholtz, 2013), production of important commodity biomolecules (Danner & Braun, 1999), production of proteins and enzyme catalysts, and production of cell-based products, including soil inoculants and food processing ingredients (Santos et al., 2019; Sybesma et al., 2017). Recent innovations in synthetic biology and genetic engineering expand the scope of possible products, such as to include advanced biofuels (Gaurav et al., 2017; Oh et al., 2018), commodity chemicals and their drop-in replacements (Burk & Van Dien, 2016), functionalized materials (Ansari & Husain, 2012), engineered cells (Becker & Wittmann 2015), and more. Specific recent examples of commercial products that leverage these advances include 1,3-propanediol by DuPont Tate and Lyle (Nakamura & Whited, 2003), 1,4-butanediol by Genomatica (Yim et al., 2011), farnesene by Amyris (Meadows et al., 2016), acetone and isopropanol by LanzaTech (Liew et al., 2022), polyethylene by Braskem (de Andrade Coutinho et al., 2013), and polylactic acid by NatureWorks, LLC (Erickson & Winters, 2012; Vink et al., 2003). Bioproducts can offer improved performance and functionality compared to existing materials (Voigt, 2020).

Bioindustrial manufacturing processes offer many advantages over strictly chemical processes and are well-positioned to address important global challenges in the coming decades (Chui et al., 2020). First, biology can be engineered to produce complex materials and substances that have properties unmatched by other manufacturing sectors. This is illustrated by the recent global COVID-19 pandemic. Bioproducts including testing supplies, treatments, and components for vaccines played an important role in the pandemic response. Materials such as polylactic acid are sufficiently robust to allow reallocation toward N95 mask production (Anon, 2020). On the horizon are more diverse biopolymers, bioplastics, and other bioproducts that have physical or chemical properties absent from the repertoire of products on the market today.

A second advantage of bioindustrial manufacturing is in its ability to reshore supply chains. The feedstocks that support bioindustrial manufacturing are diverse and include agricultural by-products, non-food crop agriculture, industrial waste streams, and gas feedstocks such as CO_2_ and methane (Clomburg et al., 2017). It is conceivable to engineer multiple bioproduction strains that convert distinct feedstocks into the same bioproduct. In this way, fluctuations in feedstock availability or price will have less of an impact on manufacturing output.

Lastly, bioindustrial manufacturing can help mitigate global environmental issues. Pivoting from a petrochemical-based economy to a bio-based economy promises to sequester carbon dioxide from the atmosphere (Scown & Keasling, 2022), reduce energy requirements for the production and transport of goods, and possibly drive a paradigm change toward smaller, distributed manufacturing facilities (Clomburg et al., 2017). Innovations in bioindustrial manufacturing will help sustain global ecosystems in the face of population growth by lowering carbon footprints, decreasing pollution, and improving renewable energy utilization efficiency. Even for products that are traditionally sourced from living organisms, like rubber or palm oil, bioindustrial manufacturing offers routes of production that are more sustainable and environmentally friendly.

## Bioindustrial Manufacturing is Different than Other Manufacturing Sectors

All manufacturing sectors face unique challenges during scale-up. There is a false narrative surrounding bioindustrial manufacturing that the ability of engineered cells to self-replicate eliminates these challenges. Scale-up of a bioindustrial manufacturing process from bench-scale to industrial-scale faces a variety of risks and unknowns, for example, the performance of a fermentation process replicated at production-relevant volumes may exhibit different behavior than predicted from much smaller volumes, depending in part on scale-down experience of the developer (Delvigne et al., 2017; Marques et al., 2010; Micheletti et al., 2006; Wang et al., 2020). Milliliter-scale fermentation vessels may not accurately reflect strain performance in kiloliter-scale vessels. Many unique challenges exist in downstream processing (DSP). Molecular intermediates and products from fermentations must typically be purified from complex aqueous media. End products of metabolic pathways can share physicochemical properties with shunt metabolites and pathway intermediates that must be purified away using sometimes tedious and expensive separation processes. However, process-guided approaches to strain engineering, coupled with application of downstream processing modeling, can mitigate such issues. For protein-based products purification, workflows often need to maintain the three-dimensional structure throughout DSP/purification so as not to lose activity (Hearn, 2017). Lastly, because the producing organisms are self-replicating, maintaining genetic stability is critical to maintaining production quality. The use of scale-down fermentation models can be used to validate genetic stability before going to scale, thus minimizing the potential impact on production scale-up.

## New Tools Are Accelerating Bioindustrial Manufacturing Workflows and Lowering R&D Costs for Early Stage Technology Development

The bioeconomy is experiencing rapid growth and has a global impact in excess of $1 trillion (Chui et al., 2020). Consistent improvement and reduction of costs for DNA synthesis and DNA sequencing are making possible new approaches in early R&D workflows that favor the massively parallel design, construction, and evaluation of hundreds to thousands of putative production strains (Esvelt et al., 2011; Smanski et al., 2014, 2016; Wang et al., 2009; Warner et al., 2010). As capabilities for rational genetic design improve, particularly the integration of machine learning capabilities, these libraries will be increasingly focused on high-performing variants (Lawson et al., 2021; Nielsen et al., 2016). New tools and approaches for genome engineering, data analysis, and learning are benefiting the bioindustrial manufacturing community. CRISPR-Cas9 enables rapid and cost-effective generation of genome modifications in virtually any organism that can be transformed with foreign DNA (Doudna & Charpentier, 2014). dCas9-based tools featuring a deactivated Cas9 enzyme provide control over gene expression and epigenetic state (Casas-Mollano et al., 2020). Base- and prime-editing tools allow for precise genome manipulation with a lower frequency of undesirable DNA repair modifications (Anzalone et al., 2020). Large-scale sequencing projects continue to provide new enzyme variants that can be exploited for metabolic engineering. Increased implementation of machine learning and artificial intelligence in biological studies is solving previously intractable problems (Jumper et al., 2021). Rapid tool advances are providing small and large companies that engineer organisms for bioindustrial manufacturing with greater precision and speed. While these innovations can accelerate ideas to the proof-of-concept stage, continued focus and innovation are needed through the pilot-, intermediate-, and industrial-scale. These innovations might be technical in nature (e.g., new fermentor designs, new membrane/resin formulations for product recovery), or they might solve problems to mitigate business risk (e.g., supply chain management and quality assurance, multi-product, or modular manufacturing plants). Manufacturing Innovation Institutes (MIIs) were established by the U.S. Government to provide the focus and funding needed to advance promising proof-of-concept technologies toward commercial readiness. BioMADE is an MII launched in early 2021 to advance bioindustrial manufacturing of non-medical products, and NIIMBL is an MII launched in 2017 with a focus on manufacturing biomedical products.

## Bioindustrial Manufacturing Poses Unique Advantages and Faces Distinct Challenges

**Fig. 1.**
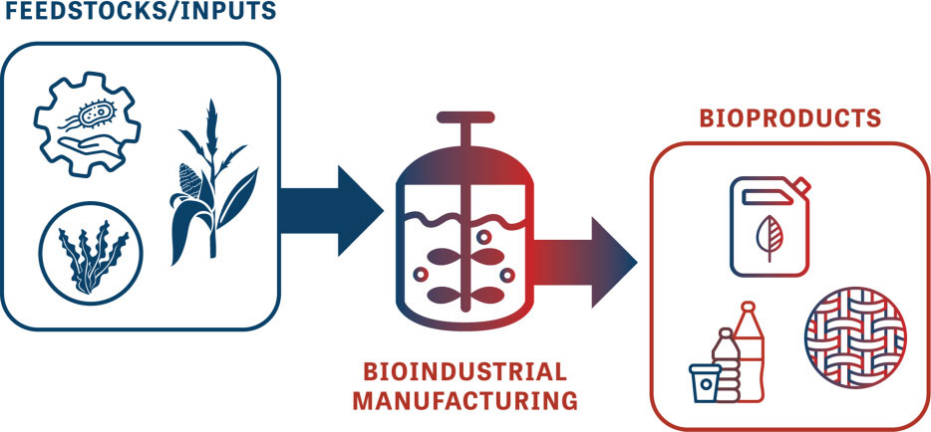
Bioindustrial manufacturing utilizes natural or engineered cells, or cell-free systems, to convert feedstocks into manufactured goods, including materials, fuels, fibers, cosmetics, food ingredients, and more.

Bioindustrial manufacturing poses several distinct advantages over conventional methods (Fig. 1). Diverse metabolic capabilities of engineered host strains lead to multiple feedstocks that can support bioindustrial manufacturing. The fraction of chemical space available to bioindustrial production is large and non-overlapping with molecules easily produced via synthetic chemistry (Wetzel et al., 2007). Enantiomerically pure products, often comprising many stereocenters, constrained ring systems, and a range of heteroatom incorporation are commonplace (Clomburg et al., 2017; Erickson & Winters, 2012). Scale-up production processes can be run under relatively mild conditions—typically at moderate temperature, low pressure, at biological pH levels, and with fewer toxic catalysts—resulting in safer, more environmentally benign processes. Perhaps the most unique advantage is the fact that engineered strains are able to grow and reproduce, which changes the economics and thought processes around R&D and industrial scale-up compared to other industries.

## Bioindustrial Manufacturing Readiness Levels (BioMRLs)

### The Readiness Levels Framework Provides a Shared Vocabulary for Confronting Risk Associated with Technology Development and Manufacturing Scale-up

The technology readiness level (TRL) system of classification was developed by NASA in the 1980s to better communicate the maturity status of a new technology (Table 1). Since then, additional field-specific readiness level frameworks have been implemented (Buchner et al., 2019; Carmack et al., 2017; Martínez-Plumed et al., 2021). In the early 2000s, leaders within the Department of Defense spearheaded the development of parallel manufacturing readiness level (MRL) classifications to cover elements of the commercialization process missing from the original TRLs. MRLs incorporate a consideration of the production costs and schedules, supply chain robustness, and pressures from competing markets, many of which change as a production process matures from lab-scale through production-relevant and production-representative environments. There is not a formal link between TRL and MRL scales, but it is not advised to mature the manufacturing readiness further than the technology readiness. Determining the readiness level for a process requires more than a quick look. The chosen level needs to be substantiated by evidence, and this evidence should be limited to demonstrated milestones (not ongoing or future planned activities). As such, it is less useful to describe a process as being ‘‘at’’ or ‘‘working on’’ MRLx—instead one can describe the technology or manufacturing process as having ‘‘attained’’, ‘‘demonstrated’’, or ‘‘achieved’’ an MRL. At the DoD and NASA, technology readiness assessments (TRAs) are performed by dedicated teams through a well-defined and rigorous process. For example, a 147-page TRA Guide was produced in 2016 by the U.S. Government Accountability Office (Government Accountability Office—GAO, 2016).

**Table 1.** Technology Readiness Levels (TRLs), paraphrased from the MRL Deskbook (http://www.dodmrl.com/MRL%20Deskbook%20V2020.pdf)

TRL	Key criterion
TRL1	Basic principles observed and reported
TRL2	Technology concept and/or application has been formulated
TRL3	R&D has commenced, and component parts of the complete system have been validated as functional
TRL4	Proof-of-concept system has been demonstrated in a laboratory environment
TRL5	Component validation in a relevant environment
TRL6	System/subsystem model or prototype demonstration in a relevant environment
TRL7	System prototype demonstration in an operational environment
TRL8	Actual system completed and qualified through test and demonstration
TRL9	Actual system proven through successful mission operations

### Translating DoD MRLs to BioMRLs

The path to commercialization for a bioindustrial manufacturing process is sufficiently distinct from other manufacturing sectors to justify the translation of a field-specific framework for BioMRLs. We describe the key criteria for each BioMRL in Table 2, and below provide more context to the nuanced differences used to distinguish between levels. In drafting this document, we frequently encountered vocabulary that is not common in the bioindustrial manufacturing literature. In these cases, we had to choose between (i) replacing vocabulary in the DoD MRLs with terms more familiar to our community or (ii) socializing the terms used in the DoD MRLs literature into our community. We did some of each and have included a glossary as Appendix B that should be referenced for key terms.

**Table 2.** Bioindustrial Manufacturing Readiness Levels (BioMRLs)

BioMRL	Summary description of BioMRL1-10
**BioMRL1:** *Basic manufacturing implications identified*	Prior to physical research and development efforts, a study of manufacturing capacity is performed. Criteria include identification and investigation of global trends in the industrial base, manufacturing science, material availability, supply chain, and metrology.
**BioMRL2:** *Manufacturing concepts identified*	Key manufacturing concepts have been identified, including broad-based studies that address analysis of material and process approaches, material effects and availability, potential supply chains, needed workforce skillsets, potential future investments, etc. Manufacturing scale and quality requirements for potential markets are identified and analyzed. An understanding of manufacturing feasibility and risk is emerging.
**BioMRL3:** *Manufacturing subsystems or components*	Components of the biomanufacturing process have been proven in a laboratory environment. This includes genetic engineering efforts needed to create strains capable of producing the desired products in titers that support the transition to pilot-scale production (typically in excess of 1 g/L). Methods for the purification and analysis of the product of interest are also required but can rely on lab-scale equipment that is not suitable for larger-scale DSP.
**BioMRL4**: *Independent validation and verification of proof-of-concept*	The proof-of-concept system has been demonstrated in a strain suitable for commercial-scale manufacturing and has been independently reproduced/validated/verified. Additionally, an initial assessment of the manufacturability is complete, including preliminary techno-economic analysis (TEA) and life-cycle analysis (LCA). This assessment should include plans for the scale-up production (SUP) and downstream processing (DSP) needed to produce sufficient quantities to allow testing and evaluation by downstream stakeholders. These plans incorporate production-relevant environments. Product quality risks and mitigation plans are documented.
**BioMRL5**: *Demonstration of prototype unit operations in a production relevant environment*	Identification of enabling/critical unit operations is complete. Prototype materials, tooling, and test equipment, as well as personnel skills, have been demonstrated empirically for unit operations in a production-relevant environment. Scale-up production and downstream processing have been performed at suitable scales to deliver sufficient quantities of end-product to downstream stakeholders for testing and evaluation. The TEA has been further refined to assess projected manufacturing costs. A risk management plan to mitigate technical and economic risks is integrated with the manufacturing strategy.
**BioMRL6**: *Demonstration of a prototype system or subsystem in a production relevant environment*	Manufacturing processes have been selected for the end-to-end manufacturing pipeline, even if engineering and/or design variables still need to be optimized. Prototype manufacturing processes and technologies, materials, tooling, and test equipment, as well as personnel skills, have been demonstrated on systems and/or subsystems in a production-relevant environment. The TEA is refined based on system performance and is expanded to include inventory control, production scheduling, plant maintenance, and production quality attributes (PQAs). Long-lead and key supply chain elements have been identified, and supply chain risk mitigation strategies exist.
**BioMRL7**: *Demonstration of systems or subsystems in a production representative environment*	Detailed system design is complete. Manufacturing processes and procedures have been demonstrated in a production representative environment. Sufficient quantities of product have been made to test packaging and distribution systems. Unit cost reduction strategies, such as statistical process controls (SPCs), are underway in a production representative environment. Quality assurance of supply chains is in place, and procurement schedules for long-lead elements are established. The manufacturing process is sufficient to support low-level commercial manufacturing.
**BioMRL8:** *Manufacturing line demonstrated, ready for low-rate initial production (LRIP)*	This maturity level is associated with manufacturing readiness for entry into LRIP. The detailed system design is complete and sufficiently stable to enter LRIP. All materials, manpower, tooling, test equipment, and facilities are proven on the manufacturing line and are available to meet the planned low-rate production schedule. STE/SIE has been validated in accordance with plans. Manufacturing and quality processes and procedures have been proven and are ready for LRIP. Known technical and business risks pose no significant challenges for LRIP. The cost model and yield and rate analyses have been updated with manufacturing line results. Supplier qualification testing and first article inspections have been completed. The industrial base has been assessed and shows that industrial capability is established to support LRIP.
**BioMRL9:** *Low rate production demonstrated; Capability in place to begin full rate production (FRP)*	Manufacturing has successfully achieved LRIP and is ready to enter FRP. All systems engineering/design requirements have been met such that there are minimal system changes. Major system design features are stable and have been proven in operational tests and evaluations. Materials, parts, manpower, tooling, test equipment, and facilities are available to meet planned rate production schedules. STE/SIE validation is maintained and re-validated as necessary. Manufacturing process capability is at an appropriate quality level to meet customer tolerances. LRIP cost targets have been met. The cost model has been updated for FRP and reflects the impact of continuous improvement.
**BioMRL10:** *FRP demonstrated and lean production practices in place*	Engineering/design changes are few and generally limited to continuous improvement changes or obsolescence issues. System, components, and items are in FRP and meet all engineering, performance, quality, and reliability requirements. Manufacturing process capability is at the appropriate quality level. All materials, tooling, inspection and test equipment, facilities, and manpower are in place and have met FRP requirements. Process infrastructure and analytical equipment validation are maintained and re-validated as necessary. Rate production unit costs meet goals, and funding is sufficient for production at the required rates. Continuous process improvements based on risks identified during FRP are ongoing.

The 10 BioMRLs can be broadly broken up into early, mid-, and late-stage maturity levels (BioMRL1-3, BioMRL4-7, and BioMRL8-10, respectively). Early stage R&D efforts needed to produce the initial proof-of-concept system are encompassed by BioMRL1-3. These MRLs can be satisfied with laboratory–environment experiments and include system-level design (BioMRL1), biomanufacturing and behavioral characterization of the required component parts (BioMRL2), or initial demonstration of a complete biomanufacturing system in laboratory environments (BioMRL3). There is a nearly one-to-one correlation between TRLs and MRLs across readiness levels, and at these early stage levels, it is arguable more important to use the TRL designation. MRLs become more important as a manufacturing process increases in scale.

Mid-stage manufacturing maturity levels are described by BioMRL4-7. For a process to achieve BioMRL4, the proof-of-concept manufacturing process should have been verified and validated, and a plan for manufacturing at scale should be articulated. Verification and validation should be done in a manner that ensures that it is sufficiently reliable (and the communication materials describing the process are sufficiently detailed) to the point that it could be confidently transferred to a new production environment with little impact on performance metrics. BioMRL5 processes have seen process unit operations demonstrated in production-relevant environments (Appendix B). This is extended in BioMRL6 to demonstrate the ability to carry out all unit operations of a manufacturing system in a production-relevant environment. Because production-relevant environments do not necessarily have all of these unit operations integrated into a single production line, it is not necessary to demonstrate complex scheduling to maximize equipment use for multi-batch production runs at this stage. BioMRL7 requires a shift from production-relevant environments to production-representative environments (Appendix B). At this stage of readiness, all unit operations should be integrated seamlessly, and the aforementioned complex scheduling can be demonstrated.

Late-stage manufacturing maturity levels include BioMRL8-10. In each of these stages, manufacturing is performed in the actual environment that will be used for industrial production. BioMRL8 is achieved when the final manufacturing line has been demonstrated and is ready for low-rate initial production (LRIP). BioMRL9 signifies that LRIP has been demonstrated and that all systems are ready to shift to full rate production (FRP). Finally, BioMRL10 is the most advanced stage of manufacturing readiness. At this level, FRP has been demonstrated, and operational changes to the manufacturing line are limited to continuous improvement changes.

## Bioindustrial Manufacturing Readiness Assessments (BioMRAs)

Assessing the maturity of a manufacturing process is done with the help of a detailed criteria matrix that describes the milestones that should be met in 25 business and technology risk categories. These risks can come from a wide range of sources, including material supply chains, manufacturing process designs, capital infrastructure, and access to a well-trained workforce. BioMRAs provide technology developers, regulators, and acquisition managers a shared process for identifying and mitigating risks in advancing manufacturing processes. Thorough and rigorous BioMRAs help to manage and communicate expectations between all stakeholders involved in bringing a product to market.

Proper BioMRAs require a non-trivial dedication of time and resources. BioMRAs should be performed by a team of subject matter experts who can identify both technical and business risks that stand in front of successful product commercialization. The BioMRLs are not aspirational states but are a set of milestones that can be justified with evidence. Well-executed BioMRAs will seek out this evidence and present it in a detailed report.

The BioMRA criteria matrix developed by this working group is included as Appendix A to this paper. BioMRAs should not be performed with a ‘‘pass/fail’’ mentality, and final assessments are expected to be nuanced. For example, the overall summary of a BioMRA could place a manufacturing process at BioMRL5, even if some of the criteria for quality management or facilities have not yet reached BioMRL5 status (presumably most of the other criteria would be at BioMRL5 or BioMRL6). Identifying the criteria that are lagging behind the general BioMRL highlights vulnerabilities to future process maturation. These vulnerabilities should be addressed before advancing the other criteria to higher maturity levels.

The BioMRA criteria matrix translates the formal criteria for MRAs available via the DoD's website (dodmrl.com) into language and examples that are accessible to the community of bioindustrial manufacturing developers, regulators, and funders. Like the BioMRLs, it does not seek to redefine target MRA milestones but merely adds nuance and context to facilitate adoption by bioindustrial manufacturing organizations. We briefly describe the primary criteria categories below that constitute rows in the MRA criteria matrix.

### Assessment Categories

These categories make up the rows of the BioMRA criteria matrix. Each category should mature continuously as a manufacturing process advances to higher maturity levels. Categories are presented in the same order as in the DoD's general MRA criteria matrix, and do not represent a listing of priority or importance.

***A. Technology and industrial base capabilities:*** Requires an analysis of the capability of the National Technology and Industrial Base (NTIB) to support the design, development, production, operation, uninterrupted maintenance support, and eventual disposal processes. The NTIB was established by Congress specifically to support national security interests, and currently supports research and development, production, maintenance, and related work in the USA, Canada, the UK, and Australia. This is an important category to consider when the DoD is a possible customer, but if the BioMRLs are used for non-materiel manufacturing processes, this category can be replaced with an assessment of the general industrial base.

***B. Design:*** Relates to the high-level design of manufacturing processes from sourcing materials, assembling unit operations in a SUP and DSP workflow, and identifying manufacturing intensification opportunities. Considerations in this category focus on system-level design and control of key characteristics of the process.

***C. Cost and funding:*** Requires an analysis of the funding needed to achieve target MRLs and milestones. This category also identifies technical and business risks that might prevent reaching manufacturing cost targets.

***D. Materials:*** Requires an analysis of the materials used in the manufacturing process, including supply chains for raw materials and the longevity/maintenance risks of materials used for manufacturing equipment and infrastructure.

***E. Bioproduction strain:*** Requires an analysis of the strain or strains used in the biomanufacturing process, including suitability for industrial biomanufacturing processes, production titers and growth considerations, genetic stability, and rules/regulations/constraints concerning strain safety, shipping, and IP rights.

***F. Process capability and control:*** Relates to the risks associated with whether manufacturing processes can reach performance levels required by the manufacturing design.

***G. Quality management:*** Requires an analysis of risks and management efforts to control quality of the final manufactured product and of the incoming supply chains and to ensure that quality is maintained as a process matures to greater manufacturing scales.

***H. Manufacturing personnel:*** Relates to assessments of the workforce to ensure that the required skills, availability, and personnel numbers are accessible to support the manufacturing effort.

***I. Facilities:*** Requires an analysis of the capabilities and capacity of key manufacturing facilities involved in a manufacturing process, including prime manufacturers, subcontractors, suppliers/vendors, and maintenance/repair providers.

***J. Manufacturing management:*** Requires an analysis of the orchestration and organization of all elements needed for a successful manufacturing process, from design to field/plant integration.

Many of the above categories are further broken down into subcategories in the BioMRA criteria matrix (Appendix A).

### Appropriately Scoping the Assessment

When biomanufacturing processes require intersecting technology development, performing an appropriately scoped TRA/MRA can become challenging. For example, imagine a project focused on developing a new analytical sensor for succinic acid that will allow for needed feedback process control for the biomanufacturing of succinic acid. There are two unique MRL maturation paths that could be considered in this case: (1) how is milestone completion on this project advancing the MRL of succinic acid biomanufacturing (in which the new sensor is an important process control element and infrastructure element) or (2) how is milestone completion on this project advancing the MRL of the new analytical sensor (which is an important consideration if you need to manufacture/sell this sensor to customers in the biomanufacturing ecosystem).

Either MRL maturation path could be important, so it is imperative to be clear at the outset of the MRA to define which path is being assessed. The two paths will not necessarily be at the same MRL at a given time. It is possible to have a fairly advanced process for succinic acid production (MRL 6/7) that does not use the sensor at all. Developing a proof-of-concept sensor and testing it in production-relevant environments (MRL4/5 for the sensor) could de-risk process control elements of succinic acid production to merit a step change in its MRL status.

We recommend that users of the BioMRA criteria matrix focus on the business model that is of primary interest to the development team. In the example above, if a team's business model is to develop, manufacture, and sell novel analytical sensors to biomanufacturing companies, then it would be better to focus a rigorous MRA on the sensor itself. Alternatively, if a team's business model is to manufacture succinic acid, and the sensor development is just an important stepping stone to enable reliable manufacturing at larger scales, then succinic acid production should be the focus of the rigorous MRA. In either case, clear communication about what manufacturing process is being assessed is important to avoid confusion.

### Interplay Between Scales and Environments

There is language within the criteria matrix below that describes experiments performed in different environments (laboratory, production-relevant, production-representative, etc.). There is an implicit correlation between these environments and the scale at which manufacturing is performed. However, each bioproduct is different, and the specific scale (e.g., in terms of liters of production medium) that is relevant for these stages in commercial readiness depends on the bioproduct and the scale-up/scale-down expertise of the developer.

Laboratory environments are inherently small-scale and include scaled-down models for production environments. They are able to predict some but not all behaviors of larger scale production runs. Laboratory-scale environments include shake flasks, small-scale (<10 L) bioreactors, and other scale-down mini-fermentation systems (e.g., Amber 250). They are commonly used in early stage research and development efforts, including an initial proof-of-concept (TRL/MRL 3). Laboratory-scale experiments, when coupled with appropriate metrology, can be invaluable for predictably scaling to production-relevant and production-representative environments. Many types of process manipulations that are feasible in laboratory environments are not feasible in production environments.

Production-relevant environments (BioMRL5/6) include pilot-scale unit operations that have process control capabilities similar to final production environments. There is no requirement to seamlessly link unit operations in production-relevant environments, and it is common that not all unit operations will be performed in the same facility at this scale. In other words, experiments that accurately test unit operations in isolation are expected at this scale.

Production-representative environments (BioMRL7) must test pilot- to intermediate-scale production runs in a facility that can seamlessly link all of the unit operations for production and DSP that will be required in the eventual commercial-scale production facility. Production-representative environments enable efficiency optimization tied to unit operation scheduling for multiple successive production runs.

### Emerging Bioindustrial Platforms

The most mature bioindustrial manufacturing platforms currently are natural or engineered microbial strain fermentations. The language used throughout this paper and the BioMRAs assumes such a platform. However, there are alternative platforms that are maturing rapidly and may feature prominently in the future bioeconomy. For example, engineered plants could be leveraged to achieve an economy of scale-up production not possible with bioreactor-based fermentations (Mizik & Gyarmati, 2021). Algal, cyanobacterial, or plant-cell culture could be utilized in innovative photosynthetic bioreactor designs to make bioproducts using CO_2_ as a feedstock and sunlight as an energy source (Deprá et al., 2019). Cell-free systems for biomanufacturing have been demonstrated at the proof-of-concept scale and could mature to compete with live-cell-based fermentations for certain products (Silverman et al., 2020). While the specific language in a BioMRA matrix would have to be tailored for some of these emerging platforms, the general considerations should hold true. For all current and future bioindustrial manufacturing platforms, mitigating manufacturing risk is best done by addressing each of the assessment categories listed above at increasingly relevant manufacturing environments and scales.

### Noteworthy Differences Between BioMRLs and Traditional MRLs

There were several modifications made to the standard DoD MRA matrix that we draw attention to here. These changes reflect recognition of the ways in which bioindustrial manufacturing differs from other manufacturing sectors. In most cases, the changes show up as new rows that were added to the BioMRA criteria matrix (e.g., bioproduction strain, measurement system maturity), but in some cases, there is merely a pivot of emphasis within an existing row (e.g., supply chain managment).

*Bioproduction strain*. The engineered strain that is grown in industrial fermentors to make a bioproduct is critically important in bioindustrial manufacturing. Milestones that pertain to the maturity of the engineered strain did not fit well into the existing criteria rows of the DoD MRA matrix. We created a new category for the bioproduction strain and further subdivided it into two subcategories: (i) chassis organism and strain characteristics and (ii) strain–environment interface.

Progression from BioMRL1 to BioMRL8, and optionally to BioMRL10 in the chassis organism and strain characteristics row begins by broadly considering potential host organisms that could be engineered to support bioproduction. These are prioritized in an analysis of alternatives (AoA) by comparing the technical risk and business risk of adopting each host into the manufacturing process. Advanced design-build-test-learn (DBTL) cycles of strain improvement focused on the strain attributes that matter for industrial scale-up are found in the low- to mid-BioMRLs. Mid- to late-BioMRL efforts focus on mitigating strain instability as well as obtaining final regulatory approval for the strain/process.

The second subcategory, strain–environment interface, has many of the same objective functions (strain growth rate and stability, bioproduct yield, titer, productivity, etc.), but focuses on understanding and de-risking the ways that bioproduction environment (e.g., fermentation medium) influences these characteristics.

*Measurement system maturity*. The ability of an experiment to reliably identify changes/improvements in key bioprocess metrics (titer, yield, productivity, etc.) is dependent on the number of replicates recorded and the measurement variation. Measurement systems that provide better precision are able to identify smaller changes in key metrics with lower numbers of replicates. Because of this, the efficiency with which an R&D team can capture knowledge throughout the BioMRL escalation ladder is impacted by the quality of measurement systems.

This is especially important for mid- and late-BioMRL experiments for two reasons. First, the desired effect size that signals process improvement becomes smaller as a process approaches maximum theoretical yield. Early in strain engineering efforts, when measured titers are only a small fraction of theoretical yield, multiple-fold improvements are possible. Low-precision (i.e., immature) measurement systems can be tolerated at this stage, since they are still capable of detecting conditions that lead to several-fold process improvements. However, processes that are already at 50% of theoretical yield *cannot* give a several-fold improvement with respect to yield alone. Instead, moderate improvements of 2–5% in yield may be the goal for R&D teams, as these can translate to millions of dollars in revenue at target manufacturing scales. High-precision measurement systems that minimize both biological and measurement variance are necessary to detect moderate improvements in bioproduction.

The second reason is that experiments become increasingly more expensive at higher BioMRLs. Advanced measurement systems that allow teams to draw statistically justified conclusions from smaller numbers of replicates allow more rapid and efficient process maturation.

For these reasons, time, effort, and funds dedicated to mitigating risks associated with measurement systems are prioritized throughout the biomanufacturing maturation timeline, and measurement system maturity is included as a new subcategory in the assessment criteria matrix. In some cases, the bioproduct material requirements for performing measurement system maturation studies during early BioMRLs are at odds with the low productivity of manufacturing processes at this stage. Improving access to and affordability of pilot-scale infrastructure to produce the first kilogram of material for testing will benefit the entire field.

*Supply chain management*. Supply chain management exists as a subcategory in the traditional MRA criteria matrix, but we seek to increase its emphasis in the BioMRA criteria matrix. Feedstock materials that constitute a critical supply chain for bioindustrial manufacturing are subject to many sources of batch variation. For domestic bioindustrial manufacturing, feedstocks may need to be procured from different hemispheres due to seasonality of agriculture. Weather/climate variation from year to year might change important aspects of feedstock quality. For these reasons, establishing a quality management system for incoming feedstocks and other important supply chain materials is arguably more important for bioindustrial manufacturing than for other manufacturing sectors.

*Pilot-line terminology*. In most cases, we have retained key terms from the DoD MRL Deskbook and translated them for the bioindustrial manufacturing community (e.g., production-relevant environment, production-representative environment, etc.) (Appendix B). However, because the use of the term ‘‘pilot-line’’ in the DoD MRL literature was commonly misinterpreted by members of our working group, we have elected to remove it from our descriptions of BioMRLs. In the DoD literature, a pilot-line environment is the final manufacturing infrastructure that will be used for LRIP and FRP (i.e., it shows up in MRL8). In contrast, the bioindustrial manufacturing community more commonly associates the terms ‘‘piloting’’ or ‘‘pilot-scale’’ with smaller-scale experiments that exist between lab-scale and intermediate scale (i.e., common for BioMRL4-5 processes). When we use the term ‘‘pilot’’ in this document, we intend to communicate the latter interpretation. We replace the usage of pilot-line in DoD literature with production-line. A production-line environment is established immediately prior to LRIP and FRP.

### Perils of R&D in the Absence of a TRL/MRL Framework

The objective functions used to guide DBTL cycles during R&D do not necessarily correspond with commercialization needs. For example, iterative DBTL cycles to improve yields for a product in a chemically defined medium will be wasteful if they do not correspond to higher yields in media suitable for commercial-scale production. In such cases, regular testing of intermediate strains in pilot-scale settings would be important to ensure that R&D of lab-scale strains does not proceed past the point of diminishing returns. Similarly, the final production titer is a convenient metric for lab-scale DBTL efforts, but the value-limiting metric at commercial scale might be the purity of that molecule relative to structurally similar metabolites (or even the concentration of one specific impurity). In such cases, there could be more value in engineering strains to decrease the production of impurities versus pushing further titer increases.

An important consideration that should be addressed early in a TRL/MRL-focused optimization is the specific host organism. Strains for which rapid and easy genetic manipulation tools are available might be ideal for early (TRL1-3) R&D but may not be well suited for the final environment of deployment, whether that is an industrial-scale bioreactor, a human gut, or a farmer's soil. Engineered multi-gene systems are known to vary in their performance between lab and industrial strains (Egbert & Klavins, 2012; Moser et al., 2012), making this transfer important early in the transition to mid-scale BioMRLs. Changes to genetic design that impact scale-up should also be made in BioMRL4. For example, the impact of plasmid-based versus chromosome-integrated systems, and the use of repetitive genetic elements that decrease genomic stability need to be addressed before advancing too far in the commercialization process (Chen et al., 2013; Englaender et al., 2017). Moving engineered systems into the final host organism early can prevent wasted DBTL effort in a strain that is not commercially viable.

**Fig. 2.**
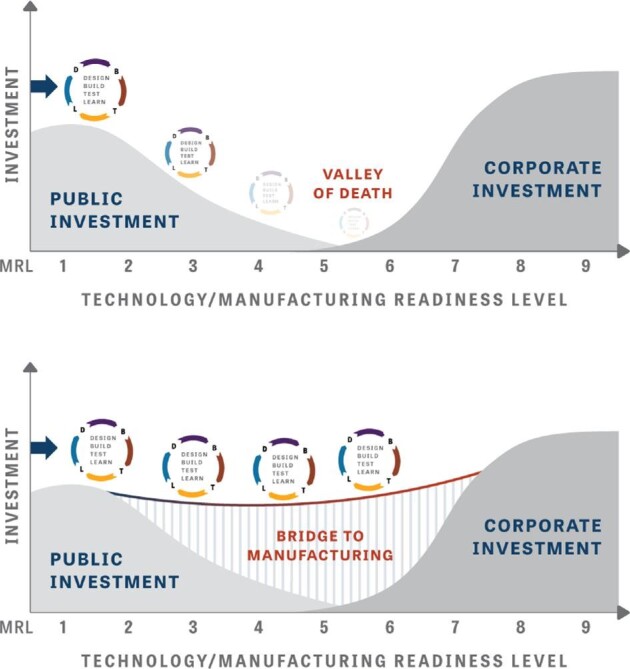
BioMRLs seek to identify and mitigate risks in scaling manufacturing processes from proof-of-concept to commercial scales. They provide a roadmap for responsible maturation of manufacturing processes.

To avoid having new technologies stall in the TRL/MRL4-7 phase, early R&D needs to be done in consideration of the limits and constraints imposed later in the scale-up process (Fig. 2). This may limit the short-term progress of TRL3-4 technology improvements, which in the absence of oversight could rapidly ‘‘improve’’ quickly down routes that in the long-term are dead ends. It is important to take the long view for applied strains and processes in parallel to or soon after demonstrating the first proof-of-concept. This will help de-risk technologies and save money over the entire course of a commercialization effort. Essentially, work with the end in mind.

## Conclusion

Bioindustrial manufacturing has special advantages and unique challenges compared to other manufacturing sectors. It can utilize diverse feedstocks, incorporating domestic supply chains to reshore manufacturing processes that have gone overseas. It can make compounds not accessible to bulk chemistry or petrochemical extraction. Producing organisms are self-replicating and can scale to large volumes when process control and measurement systems are sufficiently mature. Challenges may include more complicated DSP/purification requirements and maintaining strain stability and production efficiency. The commercialization process demands that organizations directly confront potential risks to prioritize R&D efforts. The BioMRLs framework provides a shared language for stakeholders to clearly assess the maturity toward or in commercial-scale production. Such a shared framework will help organizations to collaborate in carrying promising technologies across the ‘‘valley of death’’ from inception to commercialization. The impact of bioindustrial manufacturing on many sectors of our economy and society will continue to grow. Companies that have successfully shifted from the mindset of ‘‘scaling to learn’’ toward one of ‘‘learning to scale’’ have brought dozens of products to market in the past two decades (Hill et al., 2020), and it is exciting to see what the next two decades will bring.

## Appendix A. Bioindustrial Manufacturing Readiness Assessment Criteria Matrix

**Table:**

Category	Subcategory	BioMRL1	BioMRL2	BioMRL3	BioMRL4	BioMRL5	BioMRL6	BioMRL7	BioMRL8	BioMRL9	BioMRL10
TRL		TRL1	TRL2	TRL3	TRL4	TRL5	TRL6	TRL7	TRL7/8	TRL8/9	TRL9
A. Technology/industrial base*	A.1–Industrial base	Global trends in technology for industrial manufacturing applications were identified.	Potential industrial base capability gaps identified.	Industrial base capabilities for potential sources established for system concepts (PoC).	Industrial base capabilities surveyed for preferred materiel solutions, key technologies, components, and/or key processes. Industrial base capability risks and issues identified.	Industrial base capabilities assessment initiated to identify potential manufacturing sources. Sole/single/foreign source vendors and vendors of technologies with potential obsolescence issues identified and planning initiated to minimize risks.	Bio-industrial base capabilities assessment for MS B has been completed. Industrial capability in place to support manufacturing (mfg) of development items. Plans to minimize sole/single/foreign sources and obsolescence issues complete. Need for sole/single/foreign sources justified. Potential alternative sources identified.	Bio-industrial capability to support production analyzed. Sole/single/foreign sources stability and obsolescence issues are assessed/monitored. Potential alternate sources developed if necessary.	Bio-industrial base capability assessment for MS C completed and demonstrated in production relevant environment. Sources are available, multi-sourcing where cost-effective or necessary to mitigate risk.	Bio-industrial capability assessment for Full-Rate Production (FRP) has been completed and capability is in place to support start of FRP.	Bio-industrial capability supports Full-Rate Production (FRP) and is assessed to support modifications, upgrades, surges, and other potential manufacturing requirements.
	A.2–Manufacturing Technology Development	Global trends in manufacturing science and technology identified (i.e. concepts and capabilities).	Potential manufacturing science and technology gaps identified.	Manufacturing technology requirements established to address potential capability gaps for system concepts (PoC)	Manufacturing technology development initiatives defined for preferred materiel solution. Manufacturing technology development requirements considered to produce at an appropriate scale.	Required manufacturing technology development efforts initiated during scale-up.	Manufacturing technology efforts continuing. Required manufacturing technology development solutions demonstrated in a production relevant environment.	Manufacturing technology efforts continuing. Required manufacturing technology development solutions demonstrated in a production representative environment.	Primary manufacturing technology efforts concluding. Improvement efforts continuing. Required manufacturing technology solutions validated on a production line.	Manufacturing technology process improvement efforts initiated for FRP.	Manufacturing technology continuous process improvements ongoing.

**Table:**

Category	Subcategory	BioMRL1	BioMRL2	BioMRL3	BioMRL4	BioMRL5	BioMRL6	BioMRL7	BioMRL8	BioMRL9	BioMRL10
TRL		TRL1	TRL2	TRL3	TRL4	TRL5	TRL6	TRL7	TRL7/8	TRL8/9	TRL9
B. Design	B.1–Production Program	Conceptual designs of individual system components developed and documented. Hypotheses developed for cause–effect relationships between manufacturing variables and production.	Prototypes for various components or unit operations designed and tested individually in laboratory environments. Design variables that have a potential impact on production have been identified.	Prototype integrated system that can produce target product (cells, fermentation, downstream processing etc.) designed and tested in a laboratory environment. Work has begun on technology transfer package (TTP) design.	Initial TTP is complete. TTP describes in detail the design of all components and, for integrated system, transfer to another lab or location for independent verification and testing. Analysis of alternatives (AoA) is documented for key components/technologies.	Front-end loading 1 (FEL-1): *Conceptual Engineering Design* is complete. FEL-1 considers material and energy balance for the manufacturing process and produces a project charter. Manufacturing processes are assessed for their capability to be tested and verified in production. Process integration with Operations and Support is assessed.	FEL-2: *Basic Engineering Design* is complete. FEL-2 includes preliminary designs for equipment, facilities, and production schedule. Designs are not sufficient for construction/operation, but do allow for cost and scheduling estimates to make critical decisions that will influence the design of the plant. Trade-off studies of key technology/system component designs are complete.	FEL-3: *Detailed Engineering Design* is complete. Engineering team has a mature Systems Engineering Plan and can fully design the production plant with details on construction, commission, start-up, and operation. The proposed plant will now have a detailed cost estimate and construction schedule.	Designed production environment validated for LRIP. Data collected at commercial scale to compare against engineering design and adjustments made to detailed engineering design as appropriate.	Production design improvements analyzed for effectiveness during LRIP. Production issues and risks discovered in LRIP managed for FRP.	Production design improvements demonstrated in FRP. Production improvements ongoing. All modifications, upgrades, Diminishing Manufacturing Sources and Material Shortages (DMSMS) and other changes assessed for production.

**Table:**

Category	Subcategory	BioMRL1	BioMRL2	BioMRL3	BioMRL4	BioMRL5	BioMRL6	BioMRL7	BioMRL8	BioMRL9	BioMRL10
TRL		TRL1	TRL2	TRL3	TRL4	TRL5	TRL6	TRL7	TRL7/8	TRL8/9	TRL9
	B.2–Design Maturity	Current capability deficiencies and gaps identified.	Analyses performed to evaluate the feasibility of potential solutions to address capability gaps.	High level performance, lifecycle, and technical requirements defined and evaluated for system concepts. Trade-offs in design options that can be revealed by experiments have been identified.	Manufacturing capabilities and constraints identified for preferred systems design. SEP and T&E Strategy recognize the need for the establishment/validation of manufacturing capability and management of manufacturing risk for the product lifecycle. Initial KPPs identified for Conceptual Engineering Design, along with required measurement systems to assess KPP milestones.	Lower level performance requirements sufficient to enable Conceptual Engineering Design. All enabling/critical technologies and components identified and the product lifecycle considered. Evaluation of the design for KPPs initiated. Product data required for prototype component manufacturing is available.	Product requirements and features are sufficiently defined to support preliminary design review. Product data essential for subsystem/system prototyping is available and all enabling/critical components have been prototyped. KPPs for the detailed design identified and mitigation plans initiated.	Product design and features are sufficiently defined to support Critical Design Review (CDR) even though design change traffic may be significant. All product data essential for component manufacturing is available. Potential KPP risks and issues identified with mitigation plans in place.	Detailed design of product features and interfaces completed. All product data essential for system manufacturing available. Design change traffic does not significantly impact LRIP. KPPs are attainable based upon production line demonstrations.	Major product design features and configuration are stable. System design has been validated through operational testing of LRIP items. Design change traffic is limited. All KPPs are controlled in LRIP to appropriate quality levels.	Product design is stable. Design changes are few and generally limited to those required for continuous improvement or in reaction to obsolescence. All KPPs are controlled in FRP to appropriate quality levels.

**Table:**

Category	Subcategory	BioMRL1	BioMRL2	BioMRL3	BioMRL4	BioMRL5	BioMRL6	BioMRL7	BioMRL8	BioMRL9	BioMRL10
TRL		TRL1	TRL2	TRL3	TRL4	TRL5	TRL6	TRL7	TRL7/8	TRL8/9	TRL9
C. Cost and Funding	C.1–Production Cost Knowledge	Hypotheses developed regarding technology impact on affordability.	Cost model approach defined.	Manufacturing cost estimates for system concepts developed. Initial cost models developed which include high-level process steps and materials.	Cost estimates refined based on anticipated production volumes associated with preferred industrial solution. Cost model updated with identified cost drivers (e.g., process variables, manufacturing, materials, and special requirements). Cost driver uncertainty quantified. Cost model includes AoA.	Prototype components produced in a production relevant environment or simulations drive end-to-end cost models. Cost model includes materials, labor, equipment, infrastructure, setup, yield/scrap/rework, capability/capacity constraints.	Cost model updated with design requirements, material specifications, tolerances, integrated master schedule, results of system/subsystem simulations and production relevant prototype demonstrations.	Cost model updated with the results of systems/sub- systems produced in a production representative environment, production plant layout and design, and obsolescence solutions.	Cost models updated with results of production line build.	FRP cost model updated with result of LRIP build.	Cost model validated against actual FRP cost.
	C.2–Cost Analysis	Initial manufacturing and quality costs identified.	Potential manufacturing and quality cost drivers and system affordability gaps identified.	Analyses conducted to refine manufacturing and quality cost drivers, risks, and development strategy (e.g., lab to pilot to factory). Potential cost reduction and system affordability gap closure strategies identified.	Production and lifecycle cost risks and issues assessed for preferred industrial solution. Initial cost analysis supports Analysis of Alternatives (AoA).	Costs analyzed using prototype component actuals to ensure target costs are achievable. Decisions regarding design choices, make/buy, capacity, process capability, sources, quality, KPPs, yield/rate, and variability influenced by cost models.	Costs analyzed using prototype system/sub- system actuals to ensure target costs are achievable. Cost targets allocated to sub-systems. Cost reduction and avoidance strategies developed. Manufacturing cost drivers for ‘‘Should-Cost’’ models provided.	Mfg costs rolled up to system/sub-system level and tracked to targets. Detailed trade studies and engineering change requests supported by cost estimates. Cost reduction and avoidance strategies underway. Manufacturing cost drivers for ‘‘Should-Cost’’ models updated.	Costs analyzed using production line actuals to ensure target costs are achievable. Manufacturing cost analysis supports proposed changes to requirements or configuration. Cost reduction initiatives ongoing. Manufacturing cost drivers for ‘‘Should-Cost’’ models updated.	LRIP cost goals met and learning curve analyzed with actual data. Cost reduction initiatives ongoing. Touch labor efficiency analyzed to meet production rates and elements of inefficiency are identified with plans in place for reduction.	FRP cost goals met. Cost reduction initiatives ongoing.

**Table:**

Category	Subcategory	BioMRL1	BioMRL2	BioMRL3	BioMRL4	BioMRL5	BioMRL6	BioMRL7	BioMRL8	BioMRL9	BioMRL10
TRL		TRL1	TRL2	TRL3	TRL4	TRL5	TRL6	TRL7	TRL7/8	TRL8/9	TRL9
	C.2–Cost Analysis	Initial manufacturing and quality costs identified.	Potential manufacturing and quality cost drivers and system affordability gaps identified.	Analyses conducted to refine manufacturing and quality cost drivers, risks, and development strategy (e.g., lab to pilot to factory). Potential cost reduction and system affordability gap closure strategies identified.	Production and lifecycle cost risks and issues assessed for preferred industrial solution. Initial cost analysis supports Analysis of Alternatives (AoA).	Costs analyzed using prototype component actuals to ensure target costs are achievable. Decisions regarding design choices, make/buy, capacity, process capability, sources, quality, KPPs, yield/rate, and variability influenced by cost models.	Costs analyzed using prototype system/sub- system actuals to ensure target costs are achievable. Cost targets allocated to sub-systems. Cost reduction and avoidance strategies developed. Manufacturing cost drivers for ‘‘Should-Cost’’ models provided.	Mfg costs rolled up to system/sub-system level and tracked to targets. Detailed trade studies and engineering change requests supported by cost estimates. Cost reduction and avoidance strategies underway. Manufacturing cost drivers for ‘‘Should-Cost’’ models updated.	Costs analyzed using production line actuals to ensure target costs are achievable. Manufacturing cost analysis supports proposed changes to requirements or configuration. Cost reduction initiatives ongoing. Manufacturing cost drivers for ‘‘Should-Cost’’ models updated.	LRIP cost goals met and learning curve analyzed with actual data. Cost reduction initiatives ongoing. Touch labor efficiency analyzed to meet production rates and elements of inefficiency are identified with plans in place for reduction.	FRP cost goals met. Cost reduction initiatives ongoing.
	C.3–Manufacturing Investment Budget	Potential manufacturing investment strategy developed.	Program/projects have reasonable budget estimates for reaching MRL 3 through experimentation. Manufacturing investment budget ROM estimates identified to support industrial base and manufacturing capability gap closure strategies.	Program/projects have reasonable budget estimates for reaching MRL 4 by the milestone date. Preliminary manufacturing investment budget estimates for manufacturing gap closure recommendations have been developed.	Mfg technology initiatives incorporated to reduce costs. Program has reasonable budget estimate for reaching MRL 6 by the milestone date. Estimate includes capital investment for production relevant equipment. All outstanding MRL 4 risks and issues understood with approved mitigation plans in place.	Program has updated budget estimate for reaching MRL 6 by the milestone date. All outstanding MRL 5 risks and issues understood with approved mitigation plans in place.	Program has reasonable budget estimate for reaching MRL 8 by the milestone date. Estimate includes capital investment for production-representative equipment and production line equipment. All outstanding MRL 6 risks and issues understood with approved mitigation plans in place.	Program has updated budget estimate for reaching MRL 8 by the milestone date. All outstanding MRL 7 risks and issues understood with approved mitigation plans in place.	Program has reasonable budget estimate for reaching MRL 9 by the FRP decision point. Estimate includes investment for LRIP and FRP. All outstanding MRL 8 risks and issues understood with approved mitigation plans in place.	Program has reasonable budget estimate for FRP. All outstanding MRL 9 risks and issues understood with approved mitigation plans in place.	Production budgets sufficient for production at required rates and schedule to support funded program.

**Table:**

Category	Subcategory	BioMRL1	BioMRL2	BioMRL3	BioMRL4	BioMRL5	BioMRL6	BioMRL7	BioMRL8	BioMRL9	BioMRL10
TRL		TRL1	TRL2	TRL3	TRL4	TRL5	TRL6	TRL7	TRL7/8	TRL8/9	TRL9
D. Materials	D.1–Maturity	New material properties and characteristics surveyed and identified for research (e.g., manufacturability, quality).	Potential effects of new material properties on design application manufacturability and quality predicted based on research.	Effects of new raw material (NRM) and component properties on design concept manufacturability and quality validated using experiments.	NRMs and components for preferred industrial solution produced in a laboratory environment.	Materials manufactured or produced in a manufacturing-relevant environment. Maturation efforts in place to address new material production risks for technology demo.	Material maturity verified through technology demonstration articles. Preliminary material specifications and analytical methods in place. Material properties adequately characterized.	Material maturity sufficient for production line test. Material specifications and analytical methods approved.	Effects of NRM and component properties on design concept manufacturability and quality validated using experiments.	Materials controlled to specification in LRIP. Materials proven and validated as adequate to support FRP.	Materials controlled to specification in FRP.
	D.2–Availability	Global trends for material availability, obsolescence, and DMSMS surveyed and identified for research.	Material availability, obsolescence, and DMSMS gaps identified.	Material availability and DMSMS gap closure strategy defined.	Projected lead times identified for all difficult-to-obtain, difficult-to-process, or hazardous materials. Quantities and lead times estimated. Material availability risks and issues for preferred industrial solution considered in AoA. Mitigation plans incorporated in SEP for the preferred system concept.	Availability risks and issues addressed for prototype build. Significant material risks identified for all materials. Planning has begun to address scale-up issues.	Availability risks and issues addressed to meet process development requirements. Long-lead items identified. Components assessed for future DMSMS risk.	Availability risks and issues addressed to meet LRIP tests. Long lead procurement identified and mitigated. DMSMS mitigation strategies for components in place.	Material availability and DMSMS gap closure strategy defined.	Long-lead procurement initiated for FRP. Availability risks and issues managed for FRP.	All material availability risks and issues managed.

**Table:**

Category	Subcategory	BioMRL1	BioMRL2	BioMRL3	BioMRL4	BioMRL5	BioMRL6	BioMRL7	BioMRL8	BioMRL9	BioMRL10
TRL		TRL1	TRL2	TRL3	TRL4	TRL5	TRL6	TRL7	TRL7/8	TRL8/9	TRL9
	D.3–Supply Chain Management	Global trends for supply chain capability and capacity surveyed.	Potential supply chain capability and capacity gaps identified.	Supply chain capability and capacity gap closure strategies defined.	Survey of potential supply chain sources for preferred industrial solution completed. Supply chain capability and capacity analyses considered in the AoA.	Potential supply chain sources identified and evaluated as able to support prototype build.	Lifecycle Supply Chain requirements updated. Critical suppliers list updated. Supply chain plans in place (e.g., teaming agreements, etc.) supporting a contract award for process development.	Effective supply chain management processes defined, documented, and in place. Plan developed for predictive indicators. Assessment of critical first-tier supply chain completed (*i.e.* capability, capacity, etc.).	Supply chain capability and capacity gap closure strategies defined.	Long term agreements in place where practical. Prime's supplier management metrics (including thresholds and goals) in place and used to manage risks. Predictive indicators to manage suppliers in place. Supply chain is stable/adequate for FRP.	Supply chain proven and supports FRP requirements.
	D.4–Special Handling Requirements (shelf-life; hazards; security; etc)	Hazardous materials identified and safety procedures in place.	Initial evaluation of potential regulatory requirements and special handling concerns completed. Raw materials and components assessed for special handling and potential regulatory requirements.	EHS compliance risk for commercial scale process identified. List of hazardous materials identified and alternatives evaluated. Special handling procedures applied in the lab. Special handling concerns assessed.	EHS compliance risk for commercial scale process mitigated in lab environment. List of hazardous materials updated and alternatives assessed. Special handling procedures applied and disposal procedures evaluated. Special handling requirements identified and analyzed.	EHS requirements and special handling procedures applied in production relevant environment. Special handling requirement gaps identified. New special handling processes demonstrated in lab environment.	EHS requirements addressed and documented. Special handling procedures demonstrated in production relevant environment. Plans for special handling requirement gaps, risks, and issues complete. Manufacturing processes assessed for material storage and waste handling risks.	EHS compliance and special handling procedures demonstrated in production representative environment. Special handling procedures developed and annotated on work instructions for production line. Hazardous material storage and disposal plan in place for the production line.	EHS compliance and special handling procedures demonstrated in production line. Risks for LRIP are managed, and hazardous material storage/disposal plans are in place for LRIP.	EHS compliance demonstrated in LRIP. Special handling, and hazardous material storage and disposal procedures demonstrated in LRIP environment. Special handling, and hazardous material storage and disposal risks and issues managed for FRP.	EHS compliance demonstrated in FRP. Special handling and hazardous material storage and disposal procedures effectively implemented in FRP.

**Table:**

Category	Subcategory	BioMRL1	BioMRL2	BioMRL3	BioMRL4	BioMRL5	BioMRL6	BioMRL7	BioMRL8	BioMRL9	BioMRL10
TRL		TRL1	TRL2	TRL3	TRL4	TRL5	TRL6	TRL7	TRL7/8	TRL8/9	TRL9
E. Bioproduction Strain	E.1 Chassis organism and strain characteristics	Existing classes of chassis organisms have been assessed for their potential as bioproduction hosts.	Components or sub-systems of the designed biomanufacturing process have been engineered in a model organism related to the final production host.	Manufacturing proof of concept strain is demonstrated. Strain yield, titer, and productivity is reliably and reproducibly within an order of magnitude of required levels for commercial profitability. Freedom of use has been explored in strain variants that are suitable in for use in production-relevant environments.	Production has been demonstrated in a strain that is suitable for scale-up in production-relevant environments. Transfer rights required to share strain between pilot-, intermediate-, and commercial-scale facilities have been obtained if needed. Strain stability in production-relevant environments has been measured.	Strain performance has been assessed at pilot-manufacturing scales in production relevant environments. Required strain performance metrics, including titer, rate, yield, and stability, have been described for intermediate-scale and low-rate initial production scale. Regulatory approvals needed for use of strain have been surveyed.	Strain performance has been assessed in a production relevant environment known to be compatible with upstream and downstream process requirements. Failure-mode analysis for during-run or between-run processes (e.g., cleaning/sterilization) has been completed. Relevant regulatory considerations/consultations have been initiated.	Strain performance has been assessed in production-representative environment. Best practices for long-term strain performance reproducibility have been established in production-representative environments. Relevant regulatory approvals have been granted.	Strain performance has been assessed and is suitable for production in the biomanufacturing environment. Methods are established for maintaining strain integrity in each manufacturing batch. Opportunities for further strain improvements are recorded and pursued in parallel at lower BioMRL R&D environments. Strategies to mitigate release of proprietary information from production facility are in place (including genetic material).	Bioproduction strain is used to fulfill LRIP in the biomanufacturing environment. Logistics are in place to allow ramp-up of manufacturing to FRP. Opportunities for further strain improvements are recorded and pursued in parallel at lower BioMRL R&D environments.	Bioproduction strain is used to fulfull FRP in the biomanufacturing environment. Opportunities for further strain improvements are recorded and pursued in parallel at lower BioMRL R&D environments.
	E.2 Strain-environment interface	Options for growth and production medium have been assessed for their use in initial strain engineering and improvement workflows.	Medium variables to be targeted for growth/productivity optimization are identified for defined laboratory medium.	Medium suitable for reliable grow and production established.	Media/feedstocks suitable for production-relevant environments and scales have been identified. Growth and production traits (yield, titer, productivity) have been assessed in these media. An AoA for media and feedstocks available for commercial scale biomanufacturing has been created.	Media/feedstock supply chains and quality parameters have been identified. FMEA due to growth or bioproduction phenotypes in subtier media or feedstock components is complete.	Key parameters of media/feedstock quality that impact the performance (growth rate, yield, titer, productivity) of the bioproduction strain are known. Agreements are in place with feedstock/media providers to ensure a robust and reliable supply chain.	Feedstock/media provider regularly meets required quality levels for key parameters. A quality management system is in place to ensure that medium/feedstock availability or quality to not limit the productivity of manufacturing batches.	Availability of volume and quality of feedstock/media components is suitable to support LRIP. Feedstock/media supplier performs product Quality Testing, and acceptance testing protocols exist to verify feedstock/media testing. Plan developed for ongoing improvements/risk mitigation related to medium components.	Availability of volume and quality of feedstock/media components is suitable to support FRP. Plan in place for ongoing improvements/risk mitigation related to medium components.	Plan in place for ongoing improvements/risk mitigation related to medium components.

**Table:**

Category	Subcategory	BioMRL1	BioMRL2	BioMRL3	BioMRL4	BioMRL5	BioMRL6	BioMRL7	BioMRL8	BioMRL9	BioMRL10
TRL		TRL1	TRL2	TRL3	TRL4	TRL5	TRL6	TRL7	TRL7/8	TRL8/9	TRL9
F. Process Capability and Control	F.1–Modeling and Simulation	Scale-down models and simulation approaches and tools identified to support manufacturing and quality activities. Relevant mass and energy balances identified.	Scale-down models and simulation in development initiated. Detailed mass and energy balance equations developed.	Manufacturing and quality gaps for system concepts identified using simulation and laboratory experiments utilizing scale-down models.	Scale-down models and simulation tools utilized to define manufacturing and quality requirements for preferred materiel solution. Laboratory experiments and simulation results considered in the AoA.	Initial scale-down models and simulations (product or process) developed for each unit operation and used to determine constraints. First systems implemented to facilitate routine monitoring of mass and energy balances.	Revised scale-down models and simulations developed for each unit operation and used to refine system constraints. Initial scale-down data review and release processes utilizing control charts are formalized.	Simulations and laboratory experiments utilizing scale-down models used to determine system constraints and identify improvement opportunities.	Scale-down models and simulations verified by production line build. Results used to improve process and determine that LRIP requirements can be met.	Scale-down models and simulation verified by LRIP build, assists in management of LRIP, and determines that FRP requirements can be met.	Scale-down models and simulation verified by FRP build. Production simulation models used as a tool to assist in management of FRP.
	F.2–Manufacturing Process Maturity	Hypotheses developed regarding cause–effect relationships between process variables and process stability and repeatability. Failure Modes and Effects Analysis (FMEA) conducted to estimate Risk Priority Numbers (RPNs)	Studies performed to test high-RPN hypotheses regarding cause–effect relationships. Initial process approaches identified.	Cause–effect relationships between process control variables and process stability and repeatability validated through laboratory experiments. Critical process control variables identified.	Maturity of critical processes for preferred materiel solution assessed. Process capability requirements and improvement plans developed and documented in the SEP.	Process Maturity assessed on similar processes in production. Process capability requirements identified for production line, LRIP and FRP. FMEA revisited and RPNs revised.	Manufacturing processes demo'd in production relevant environment. Collection or estimation of process capability data from prototype build and refinement of process capability requirements initiated. Work to mitigate high-RPN failure modes ongoing.	Manufacturing processes demonstrated in a production representative environment. Collection and/or estimation of process capability data and refinement of process capability requirements ongoing. Work to mitigate high-RPN failure modes concluded.	Manufacturing processes for LRIP verified on a production line. Process Capability data from production line meets target. Process capability requirements for LRIP and FRP refined based upon production line data.	Manufacturing processes are stable, adequately controlled, capable, and have achieved program LRIP objectives. Variability experiments conducted to show FRP impact and potential for continuous improvement.	Manufacturing processes are stable, adequately controlled, capable, and have achieved program FRP objectives.

**Table:**

Category	Subcategory	BioMRL1	BioMRL2	BioMRL3	BioMRL4	BioMRL5	BioMRL6	BioMRL7	BioMRL8	BioMRL9	BioMRL10
TRL		TRL1	TRL2	TRL3	TRL4	TRL5	TRL6	TRL7	TRL7/8	TRL8/9	TRL9
	F.3–Process Yields and Rates	Hypotheses developed regarding future state manufacturing yields and rates.	Studies performed to test hypotheses regarding yields and rates.	Initial estimates of yields and rates for system concepts identified through laboratory experiments. Yield and rate gaps for system concepts identified.	Yield and rate assessments on preferred materiel solution completed and considered in the AoA. Yield and rate gap closure strategies identified for the preferred materiel solution and documented in the SEP.	Target yields and rates established for production line, LRIP, and FRP. Yield and rate issues identified. Improvement plans developed/initiated.	Yields and rates from production relevant environment evaluated against targets and the results feed improvement plan.	Yields and rates from production representative environment evaluated against production line targets and the results feed improvement plans.	production line targets achieved. Yields and rates required to begin LRIP refined using production line results. Improvement plans ongoing and updated.	LRIP yield and rate targets achieved. Yields and rates required to begin FRP refined using LRIP results. Yield improvements ongoing.	FRP yield and rate targets achieved. Yield improvements on-going.
	F.4–Measurement system maturity	Concepts developed for test method controls. Availability of reference materials and standards (broadly defined) has been assessed.	Initial reference materials are purchased and/or prepared. First estimates of measurement method repeatability and reproducibility are propagated through yield and rate formulae, and through detailed mass balance equations.	Initial measurement method precision targets specified. Measurement method capability and performance are evaluated against targets. Measurement improvement plan is described.	Measurement method precision targets refined based on initial estimates of laboratory process variation. First systems implemented to facilitate routine monitoring and quantification of measurement method capability, performance and stability.	Measurement method precision targets refined based on yield, rate and mass balance issues identified. Initial data review and release processes utilizing control charts are formalized.	Measurement method precision targets refined based on narrowing gap between target yields and rates and demonstrated outcomes. Improvements to measurement methods, reporting systems and data review processes ongoing.	Contribution of measurement methods (for titer, rate, and yield) to total manufacturing process variation demonstrated to be <1/3 of observed standard deviation. Precision-to-tolerance ratios for all methods used to characterize production demonstrated to be <10%.	Measurement method precision targets refined using production line results. All measurement method stabilities demonstrated to be <1.33. Improvements to measurement methods, reporting systems and data review processes ongoing.	Measurement method precision targets refined using LRIP results. Improvements to Measurement methods, reporting systems and data review processes ongoing.	Measurement method precision targets refined using FRP results. Improvements to measurement methods, reporting systems and data review processes ongoing.

**Table:**

Category	Subcategory	BioMRL1	BioMRL2	BioMRL3	BioMRL4	BioMRL5	BioMRL6	BioMRL7	BioMRL8	BioMRL9	BioMRL10
TRL		TRL1	TRL2	TRL3	TRL4	TRL5	TRL6	TRL7	TRL7/8	TRL8/9	TRL9
G. Quality Risk Management	G.1–Quality Management	Quality management considerations surveyed and included in early planning activities.	Quality management needs assessed, analyzed, and validated.	Quality management requirements for system concepts identified.	Quality strategy for the preferred materiel solution developed, considered in the AoA, and documented in the SEP.	Quality strategy updated to reflect KPP identification activities.	Initial Quality Plan and Quality Management System (QMS) is in place. Quality risks and metrics have been identified and initiated.	Quality targets established. QMS elements (e.g., control of nonconforming material, corrective action, etc.) meet requirements of appropriate industry standards. Program specific Quality Program Plan being developed.	Program-specific Quality Program Plan established. Program Quality Manager assigned. Quality targets assessed against production line, results feed continuous quality improvements.	Quality targets verified on LRIP line. Continuous quality improvement ongoing. Management review of Quality measures conducted on regular basis and appropriate actions taken.	Quality targets verified on FRP line. Continuous quality improvement ongoing. Statistical controls applied where appropriate.
	G.2–Product Quality	Quality metrology state of the art surveyed. Hypotheses developed regarding cause–effect relationships between technology variables and quality.	Studies performed to test hypotheses regarding cause–effect relationships between technology variables and quality. Elements (i.e. materials, processes, capabilities, limitations) identified which have a potential impact on product quality attributes (PQAs).	System concept elements evaluated for quality using experiments and simulations. Initial PQA requirements, risks, and issues identified. Product quality measurement systems identified.	PQA requirements and the inspection and acceptance testing strategy for the preferred solution considered in AoA. Product quality risk and issue mitigation plans documented in the SEP.	Roles and responsibilities identified for acceptance test procedures, in-process and final inspections, and SPCs for prototype units.	KPP management approach defined. Initial requirements identified for acceptance test procedures and in-process and final inspection requirements for commercial scale units. Appropriate inspection and acceptance test procedures identified for prototype units.	Quality data from the production representative environment collected and analyzed and results used to shape improvement plans. Control plans completed for management of KPPs. Test and Inspection plans being developed for EMD units.	KPPs managed. Measurement procedures and controls in place (e.g., SPC, FRACAS, audits, customer satisfaction, etc.). Production line data meets capability requirements for all Key Characteristics. Test and Inspection plans complete and validated for production units.	Data from LRIP demonstrates production processes for all KPPs and other manufacturing processes critical to quality, are capable and under control for FRP.	KPPs controlled at FRP. Results achieve targeted statistical level on all KPPs. Results reflect continuous improvement.

**Table:**

Category	Subcategory	BioMRL1	BioMRL2	BioMRL3	BioMRL4	BioMRL5	BioMRL6	BioMRL7	BioMRL8	BioMRL9	BioMRL10
TRL		TRL1	TRL2	TRL3	TRL4	TRL5	TRL6	TRL7	TRL7/8	TRL8/9	TRL9
	G.3–Supplier Quality Management	Supplier quality and quality management systems state of the art surveyed.	Initial supplier quality and quality management systems evaluated.	Supplier quality and quality management system requirements for unit operations identified.	Potential supplier quality capabilities, risks, and issues identified for the preferred materiel solution, including sub-tier suppliers. Supplier quality management system requirements defined, and documented.	Supply base quality capabilities and risks identified, including sub-tier supplier quality management.	Supply base quality improvement initiatives identified addressing supplier QMS shortfalls, including subtier supplier quality management.	Key supplier QMSs) meet appropriate industry standards. Supplier quality data from production representative units collected and analyzed. Strategy for audits of critical supplier processes outlined.	Supplier program-specific QMSs are adequate. Supplier products qualification testing and first article inspection completed. Acceptance testing of supplier products is adequate to begin LRIP. Plan for subcontractor process audits in place and implemented by prime contractor.	Supplier quality management of KPPs and other critical manufacturing processes demonstrates capability and control for FRP. Acceptance testing of supplier products reflects control of quality adequate to begin FRP. Subcontractor quality audits performed as necessary to ensure subcontractor specification compliance.	Supplier quality data reflects adequate management of KPPs and control of critical manufacturing processes, including quality management down to sub-tier suppliers. Results achieve high statistical level (e.g., 6-sigma) on all critical dimensions. Subcontractor quality audits performed as necessary to ensure subcontractor specification compliance
H. Mfg. Workforce	H.1.—Mfg Workforce	Workforce skillsets to support emerging trends in manufacturing and technology surveyed.	Workforce skillsets to support emerging trends in manufacturing and technology evaluated.	Workforce skillset requirements for system concepts identified. Workforce skillset capabilities gaps identified.	Workforce skillset and production workforce requirements (technical and operational) for the preferred material solution identified and considered in the AoA. Workforce training and development requirements to close skillset gaps defined. Availability of workforce for process development/maturation determined.	Skillsets identified and plans developed to meet prototype and production needs. Special skills certification and training requirements established.	Manufacturing workforce skills available for production in a relevant environment. Resources (quantities and skillsets) identified and initial plans developed to achieve requirements for production line and LRIP.	Manufacturing workforce resource requirements identified and plans developed to achieve LRIP requirements. LRIP personnel trained on production line where possible. Plans to achieve FRP workforce requirements initiated based on production line.	LRIP personnel requirements met. Plan to achieve FRP workforce requirements implemented.	FRP personnel requirements met. Production workforce skill sets maintained in spite of workforce attrition.	

**Table:**

Category	Subcategory	BioMRL1	BioMRL2	BioMRL3	BioMRL4	BioMRL5	BioMRL6	BioMRL7	BioMRL8	BioMRL9	BioMRL10
TRL		TRL1	TRL2	TRL3	TRL4	TRL5	TRL6	TRL7	TRL7/8	TRL8/9	TRL9
I. Facilities	I.1–Process infrastructure and Analytical Equipment	State of the art process infrastructure and analytical equipment surveyed.	Potential process infrastructure and analytical equipment needed by this process is being considered.	Process infrastructure and analytical equipment that is available has been identified. Gaps in the available process infrastructure and analytical equipment that need to be filled for your technology to mature have been identified.	An AoA has been performed, describing the landscape of possible process infrastructure and analytical equipment that will be available for manufacturing. The preferred process infrastructure and analytical equipment options have been identified.	The preferred process infrastructure and analytical equipment options are supported with rationale and a schedule for procurement.	Process infrastructure and analytical equipment has been demonstrated in a production relevant environment using material manufactured at pilot-scales. Any technological improvements to process infrastructure and analytical methods needed for incorporation to a manufacturing environment are complete.	Process infrastructure and analytical equipment has been validated in production representative environment Development or procurement of the process infrastructure and analytical equipment needed for LRIP is initiated. Maintenance strategy for process infrastructure and analytical equipment that will prevent down-time in manufacturing has been developed.	Process infrastructure and analytical equipment has been demonstrated in production-line environment, and modifications needed to support LRIP have been identified. Equipment maintenance has been demonstrated on the production-line.	All process infrastructure and analytical equipment has been proven effective for low-rate industrial production. Requirement for scaling to full industrial production have been identified. Manufacturing equipment maintenance schedule has been demonstrated.	Process infrastructure and analytical equipment needed to support full rate production has been demonstrated. Planned maintenance schedule has been achieved.
	I. 2–Facilities	Current facility capabilities and capacity at your organization (or available to your organization) have been surveyed.	Potential facility capability and capacity requirements identified.	Gaps between required facility capabilities and capacities identified.	Facilities with the capability (unit operations) and capacity to meet prototype production have been identified. This should include an Analysis of Alternatives. Human factors (ergonomics and safety systems) of the facility have been identified.	Manufacturing facilities needed for prototype production are demonstrated. Human factors (ergonomics and safety) during prototype production are addressed.	Facility capabilities and capacity needed for production-line development are developed. Human factors (ergonomics and safety systems) are demonstrated in a production relevant environment.	Facility capabilities and capacity needed for LRIP identified. Human factors (ergonomics and safety) are demonstrated in production representative environment.	Production-line facilities are demonstrated. Facilities are adequate to support LRIP. Plans are in place to transition to FRP. Workplace safety is adequate. Human factors (ergonomics and safety systems) are demonstrated on production line.	Facilities are in place to demonstrate LRIP. Capacity plans adequate to support FRP. Human factors & ergonomics and safety practices for manufacturing (personnel, processes & equipment) demonstrated in LRIP.	Production facilities in place and capacity demonstrated to meet maximum FRP requirements. Human factors & ergonomics and safety requirements for manufacturing (personnel, processes & equipment) demonstrated in FRP.

**Table:**

Category	Subcategory	BioMRL1	BioMRL2	BioMRL3	BioMRL4	BioMRL5	BioMRL6	BioMRL7	BioMRL8	BioMRL9	BioMRL10
TRL		TRL1	TRL2	TRL3	TRL4	TRL5	TRL6	TRL7	TRL7/8	TRL8/9	TRL9
J. Mfg. Management	J.1–Mfg Planning & Scheduling	Manufacturing management considerations surveyed and included in early planning activities.	Manufacturing management needs assessed, analyzed and validated.	Manufacturing management requirements for system concepts identified.	Manufacturing strategy for the preferred solution developed, considered in the Analysis of Alternatives (AoA), and documented in the Acquisition Strategy (AS). Prototype schedule risk mitigation efforts documented in the Systems Engineering Plan.	Manufacturing strategy refined based upon preferred concept. Prototype schedule risk mitigation efforts initiated.	Initial manufacturing approach developed. All system design related mfg events included in Integrated Master Plan/Schedule (IMP/IMS). Manufacturing risk, and issue mitigation approach for production line and/or technology insertion programs defined.	Initial Manufacturing Plan developed and included in IMP/IMS. Manufacturing risks and issues integrated into mitigation plans. Initial work instructions developed. Effective production control system in place to support production line.	Manufacturing Plan updated for LRIP. All manufacturing risks and issues identified and assessed with approved mitigation plans in place. Work instructions finalized. Effective production control system in place to support LRIP.	Manufacturing plan updated for FRP. All manufacturing risks and issues managed. Effective production control system in place to support FRP.	All manufacturing risks and issues managed.
	J.2 Materials Planning	Materials planning state of the art surveyed.	Initial availability, lead time, handling and storage requirements for potential materials and components evaluated.	Materials and components list for system concepts developed. Initial materials planning requirements (i.e. availability, lead times, handling, and storage) identified.	Materials and components list with estimates for availability, lead times, handling and storage requirements developed and considered in the AoA.	Make/buy evaluations initiated and include production considerations for production line, LRIP, and FRP needs. Lead times and other materials risks and issues identified.	Most material make/buy decisions complete, material risks and issues identified, and mitigation plans developed. Bill of Materials (BOM) initiated.	Make/Buy decisions and BOM complete for production line build. Material planning systems in place for production line build.	Make/Buy decisions and BOM complete to support LRIP. Material planning systems proven on production line for LRIP build.	Make/Buy decisions and BOM complete to support FRP. Material planning systems proven in LRIP and sufficient for FRP.	Material planning systems validated on FRP build.

## Appendix B. Glossary of Key Terms

**Acquisition strategy (AS):** Describes the Program Manager's plan to achieve program execution and programmatic goals across the entire program life cycle. Summarizes the overall approach to acquiring the capability (including the program schedule, structure, risks, funding, and business strategy). Contains sufficient detail to allow senior leadership and the milestone decision authority (MDA) to assess whether the strategy makes good business sense, effectively implements laws and policies, and reflects management's priorities. Once approved by the MDA, the acquisition strategy provides a basis for more detailed planning. The strategy evolves over time and should continuously reflect the current status and desired goals of the program.

**Analysis of alternatives (AoA)**: From Defense Acquisition Guidebook Ch2-2.3 (for detailed definitions of Milestones A, B, and C in this context, please see the Guidebook): The AoA is an important element of the defense acquisition process. An AoA is an analytical comparison of the operational effectiveness, suitability, and life-cycle cost of alternatives that satisfy established capability needs. After the Materiel Development Decision, the AoA is initiated to examine potential materiel solutions with the goal of identifying the most promising option, thereby guiding the Materiel Solution Analysis phase. Subsequently, an update to the AoA is initiated when necessary or mandated by the DAE at the start of the Technology Maturation and Risk Reduction phase and is reviewed at Milestone B (which usually represents the first major funding commitment to the acquisition program). The update to the AoA is used to refine the proposed materiel solution as well as to reaffirm the rationale, in terms of cost-effectiveness, for initiation of the program into the formal systems acquisition process. For major defense acquisition programs at Milestone A, the MDA must certify in writing to the Congress that the Department has completed an AoA consistent with the study guidance developed by the Director, Cost Assessment and Program Evaluation (DCAPE), in addition to meeting other certification criteria. For major defense acquisition programs at Milestone B, the MDA must certify in writing to Congress that the Department has completed an AoA with respect to the program, in addition to meeting other certification criteria. Pursuant to DoDI 5000.02, the AoA is updated as needed at Milestone C.

**Diminishing manufacturing sources and material shortages (DMSMS):** The loss or impending loss of manufacturers of items or suppliers of items or raw materials, also known as obsolescence.

**Environment, health, and safety (EHS):** An acronym for the set that studies and implements the practical aspects of protecting the environment and maintaining health and safety at job sites and in places where the manufactured product is sold/used.

**Failure modes and effects analysis (FMEA):** A formal process of reviewing the manufacturing system and subsystems to identify the potential failure modes, as well as their causes and effects. The failure modes of each component are organized in an FMEA worksheet, for which several templates can be found online. FMEAs should define the probability (P), severity (S), and means of detection (D) of each failure mode. From these metrics, it is possible to determine a risk level for each failure mode.

**Failure reporting, analysis, and corrective system (FRACAS):** A closed-loop system for performing failure mode analysis and correction.

**Front-end loading (FEL):** An approach to feasibility analysis or conceptual planning commonly used for stage-gating in manufacturing industries. It involves gathering strategic information at process-development milestones to mitigate risk in process scale-up.

**Full-rate production (FRP):** A manufacturing process that has previously been demonstrated at LRIP and is scaled to address the required market demand.

**Key performance parameters (KPPs):** Performance attributes of a system considered critical to the development process. These can be staged to increase during the course of manufacturing process maturity. They should be linked to empirical measurements that facilitate objective assessment.

**Low-rate initial production (LRIP):** LRIP is the initial phase of product manufacturing and sales to customers. LRIP is meant to be temporary and is intended to allow for information collection on all aspects of the commercial-scale manufacturing process prior to FRP. The duration of LRIP should be minimal and sufficient to permit identification and resolution of any deficiencies prior to FRP. LRIP does not necessarily have to be an economic stand-alone rate of production, but it will ensure that transition to FRP is as economical as possible.

**Modeling versus simulation:** For the purpose of this document, we refer to models as scaled-down physical systems meant to capture key behaviors of biomanufacturing processes. Modeling in this sense includes lab-scale (flask and smaller) fermentations, lab-scale apparatus to allow for empirical measurements of DSP unit operations (e.g., extraction efficiencies), and others. Computational modeling is captured in the term ‘‘simulations’’.

**New raw material (NRM):** A raw material that is not currently commercially available in quantities needed.

**Product quality attributes (PQA):** The combination of attributes including performance features and characteristics of a product that impact a customer's satisfaction/acceptance.

**Quality management systems (QMSs):** A collection of business processes focused on achieving quality policy and quality objectives to meet customer requirements.

**Risk priority number (RPN):** A quantitative metric of a particular failure mode. The RPN is the product of a failure mode's probability (P), severity (S), and ease of detection (D) (low numbers signifying easy detection and high numbers signifying difficult detection). Greater RPNs indicate higher risk.

**Strain:** A clonal lineage of a natural or engineered cell that self-replicates. Sometimes this term is used interchangeably with cell-line or production host.

**Statistical process control (SPC):** An approach for quality control that uses statistical methods to monitor and control a manufacturing process, emphasizing early detection/correction as preventative measures to ensure product quality rather than observing quality defects after they have occurred.

**Systems engineering plan (SEP)**: SEP in the DoD MRL Deskbook is equivalent to the FEL-3 *Detailed Engineering Design* of the front-end loading process. It includes a fully designed production plant, including the exact specifications for construction, commission, start-up, and operation.

**Technology transfer package (TTP):** A document or set of documents that describes the new technology (product or process) so as to facilitate its transfer to another group. TTPs are required, for example, prior to working with a contract manufacturing organization or industrial production partner. They should contain all of the required information for the partner organization to plan, allocate resources, and successfully execute on the manufacturing objective.

## References

[bib2] Anon . (2020). NatureWorks partners with Nonwovens Institute to support production of 10 million N95 masks for healthcare workers fighting COVID-19. BusinessWire.

[bib3] Ansari S. A. , Husain Q. (2012). Potential applications of enzymes immobilized on/in nano materials: a review. Biotechnology Advances, 30(3), 512–523. 21963605 10.1016/j.biotechadv.2011.09.005

[bib4] Anzalone A. V. , Koblan L. W., Liu D. R. (2020). Genome editing with CRISPR–Cas nucleases, base editors, transposases and prime editors. Nature Biotechnology, 38(7), 824–844. 10.1038/s41587-020-0561-9 32572269

[bib5] Becker J. , Wittmann C. (2015). Advanced biotechnology: metabolically engineered cells for the bio-based production of chemicals and fuels, materials, and health-care products. Angewandte Chemie International Edition, 54(11), 3328–3350. 25684732 10.1002/anie.201409033

[bib6] Buchner G. A. , Stepputat K. J., Zimmermann A. W., Schomäcker R. (2019). Specifying technology readiness levels for the chemical industry. Industrial and Engineering Chemistry Research, 58(17), 6957–69.

[bib7] Burk M. J. , Van Dien S. (2016). Biotechnology for chemical production: challenges and opportunities. Trends in Biotechnology, 34(3), 187–190. 26683567 10.1016/j.tibtech.2015.10.007

[bib8] Carmack W. J. , Braase L. A., Wigeland R. A., Todosow M. (2017). Technology readiness levels for advanced nuclear fuels and materials development. Nuclear Engineering and Design, 313, 177–184.

[bib9] Casas-Mollano J. A , Zinselmeier M. H., Erickson S. E., Smanski M. J. (2020). CRISPR-Cas activators for engineering gene expression in higher eukaryotes. The CRISPR Journal, 3(5), 350–364. 33095045 10.1089/crispr.2020.0064 PMC7580621

[bib10] Chen Y.-J. , Liu P., Nielsen A. A. K., Brophy J. A. N., Clancy K., Peterson T., Voigt C. A. (2013). Characterization of 582 natural and synthetic terminators and quantification of their design constraints. Nature Methods, 10(7), 659–664. 23727987 10.1038/nmeth.2515

[bib11] Chui M. , Evers M., Manyika J., Zheng A., Nisbet T. (2020). The bio revolution: Innovations transforming economies, societies, and our lives. McKinsey Global Institute.

[bib12] Clomburg J. M. , Crumbley A. M., Gonzalez R. (2017). Industrial biomanufacturing: The future of chemical production. Science, 355(6320), aag0804. 28059717 10.1126/science.aag0804

[bib13] Danner H. , Braun R. (1999). Biotechnology for the production of commodity chemicals from biomass. Chemical Society Reviews, 28(6), 395–405.

[bib14] Delvigne F. , Takors R., Mudde R., Gulik W., Noorman H. (2017). Bioprocess scale-up/down as integrative enabling technology: From fluid mechanics to systems biology and beyond. Microbial Biotechnology, 10(5), 1267–1274. 28805306 10.1111/1751-7915.12803 PMC5609235

[bib1] de Andrade Coutinho P. L. , Morita A. T., Cassinelli L. F., Morschbacker A., Werneck Do Carmo R. (2013). Braskem's ethanol to polyethylene process development. Catalytic Process Development for Renewable Materials. (149–165). Wiley.

[bib15] Deprá M. C. , Mérida L. G. R., de Menezes C. R., Zepka L. Q., Jacob-Lopes E. (2019). A new hybrid photobioreactor design for microalgae culture. Chemical Engineering Research and Design, 144, 1–10.

[bib16] Doudna J. A. , Charpentier E. (2014). The new frontier of genome engineering with CRISPR-Cas9. Science, 346(6213), 1258096. 25430774 10.1126/science.1258096

[bib17] Egbert R. G. , Klavins E. (2012). Fine-tuning gene networks using simple sequence repeats. Proceedings of the National Academy of Sciences of the United States of America, 109(42), 16817–16822. 22927382 10.1073/pnas.1205693109 PMC3479488

[bib18] Englaender J. A. , Jones J. A, Cress B. F., Kuhlman T. E., Linhardt R. J., Koffas M. A. G. (2017). Effect of genomic integration location on heterologous protein expression and metabolic engineering in *E. Coli*. ACS Synthetic Biology, 6(4), 710–720. 28055177 10.1021/acssynbio.6b00350

[bib19] Erickson B. , Winters P. (2012). Perspective on opportunities in industrial biotechnology in renewable chemicals. Biotechnology Journal, 7(2), 176–185. 21932250 10.1002/biot.201100069 PMC3490365

[bib20] Esvelt K. M. , Carlson J. C., Liu D. R. (2011). A system for the continuous directed evolution of biomolecules. Nature, 472(7344), 499–503. 21478873 10.1038/nature09929 PMC3084352

[bib21] Gaurav N. , Sivasankari S., Kiran G. S., Ninawe A., Selvin J. (2017). Utilization of bioresources for sustainable biofuels: a review. Renewable and Sustainable Energy Reviews, 73, 205–214.

[bib22] Government Accountability Office - GAO . (2016). GAO Technology Readiness Assessment Guide: Best Practices for Evaluating the Readiness of Technology for Use in Acquisition Programs and Projects -Exposure Draft. (GAO-16-410G). 147.

[bib23] Hearn M. T. W. (2017). Recent progress toward more sustainable biomanufacturing: Practical considerations for use in the downstream processing of protein products. Preparative Chromatography for Separation of Proteins 537–582.

[bib24] Hill P. , Benjamin K., Bhattacharjee B., Garcia F., Leng J., Liu C-Li, Murarka A., Pitera D., Porcel E. M. R., da Silva I. (2020). Clean Manufacturing powered by biology: How Amyris has deployed technology and aims to do it better. Journal of Industrial Microbiology & Biotechnology: Official Journal of the Society for Industrial Microbiology and Biotechnology, 47(11), 965–975. 10.1007/s10295-020-02314-3 PMC7695652 33029730

[bib25] Hugenholtz J. (2013). Traditional biotechnology for new foods and beverages. Current Opinion in Biotechnology, 24(2), 155–159. 23395405 10.1016/j.copbio.2013.01.001

[bib26] Jumper J. , Evans R., Pritzel A., Green T., Figurnov M., Ronneberger O., Tunyasuvunakool K., Bates R., Žídek A., Potapenko A. (2021). Highly accurate protein structure prediction with AlphaFold. Nature, 596(7873), 583–589. 34265844 10.1038/s41586-021-03819-2 PMC8371605

[bib27] Lawson C. E. , Martí J. M., Radivojevic T., Jonnalagadda S. V. R., Gentz R., Hillson N. J., Peisert S., Kim J., Simmons B. A., Petzold C. J. (2021). Machine learning for metabolic engineering: A review. Metabolic Engineering, 63, 34–60. 33221420 10.1016/j.ymben.2020.10.005

[bib28] Liew F. E. , Nogle R., Abdalla T., Rasor B. J., Canter C., Jensen R. O., Wang L., Strutz J., Chirania P., De Tissera S. (2022). Carbon-negative production of acetone and isopropanol by gas fermentation at industrial pilot scale. Nature Biotechnology, 40(3), 335–344. 10.1038/s41587-021-01195-w 35190685

[bib29] Marques M. P. C. , Cabral J. M. S., Fernandes P. (2010). Bioprocess scale-up: Quest for the parameters to be used as criterion to move from microreactors to lab-scale. Journal of Chemical Technology & Biotechnology, 85(9), 1184–1198.

[bib30] Martínez-Plumed F. , Gómez E., Hernández-Orallo J. (2021). Futures of artificial intelligence through technology readiness levels. Telematics and Informatics, 58, 101525.

[bib31] Meadows A. L. , Hawkins K. M., Tsegaye Y., Antipov E., Kim Y., Raetz L., Dahl R. H., Tai A., Mahatdejkul-Meadows T., Xu L. (2016). Rewriting yeast central carbon metabolism for industrial isoprenoid production. Nature, 537(7622), 694–697. 27654918 10.1038/nature19769

[bib32] Micheletti M. , Barrett T., Doig S. D., Baganz F., Levy M. S., Woodley J. M., Lye G. J. (2006). Fluid mixing in shaken bioreactors: Implications for scale-up predictions from microlitre-scale microbial and mammalian cell cultures. Chemical Engineering Science, 61(9), 2939–2949.

[bib33] Mizik T. , Gyarmati G. (2021). Economic and sustainability of biodiesel production—a systematic literature review. Clean Technologies, 3(1), 19–36.

[bib34] Moser F. , Broers N. J., Hartmans S., Tamsir A., Kerkman R., Roubos J. A., Bovenberg R., Voigt C. A. (2012). Genetic circuit performance under conditions relevant for industrial bioreactors. ACS Synthetic Biology, 1(11), 555–564. 23656232 10.1021/sb3000832 PMC3904225

[bib35] Nakamura C. E. , Whited G. M. (2003). Metabolic engineering for the microbial production of 1, 3-propanediol. Current Opinion in Biotechnology, 14(5), 454–459. 14580573 10.1016/j.copbio.2003.08.005

[bib36] Nielsen A. A. K. , Der B. S., Shin J., Vaidyanathan P., Paralanov V., Strychalski E. A., Ross D., Densmore D., Voigt C. A. (2016). Genetic circuit design automation. Science, 352(6281), 53–64. 10.1126/science.aac7341 27034378

[bib37] Oh Y.-K. , Hwang K.-R., Kim C., Kim J. R., Lee J.-S. (2018). Recent developments and key barriers to advanced biofuels: A short review. Bioresource Technology, 257, 320–333. 29523378 10.1016/j.biortech.2018.02.089

[bib38] Santos M. S. , Nogueira M. A., Hungria M. (2019). Microbial inoculants: Reviewing the past, discussing the present and previewing an outstanding future for the use of beneficial bacteria in agriculture. Amb Express, 9(1), 1–22. 31865554 10.1186/s13568-019-0932-0 PMC6925611

[bib39] Scown C. D. , Keasling J. D. (2022). Sustainable manufacturing with synthetic biology. Nature Biotechnology, 40(3), 304–307. 10.1038/s41587-022-01248-8 35190687

[bib40] Silverman A. D. , Karim A. S., Jewett M. C. (2020). Cell-free gene expression: An expanded repertoire of applications. Nature Reviews Genetics, 21(3), 151–170. 10.1038/s41576-019-0186-3 31780816

[bib41] Smanski M. J. , Bhatia S., Zhao D., Park Y. J., Woodruff L. B. A., Giannoukos G., Ciulla D., Busby M., Calderon J., Nicol R., Benjamin Gordon D., Densmore D., Voigt C. A. (2014). Functional optimization of gene clusters by combinatorial design and assembly. Nature Biotechnology, 32(12), 1241–1249. 10.1038/nbt.3063 25419741

[bib42] Smanski M. J. , Zhou H., Claesen J., Shen B., Fischbach M. A., Voigt C. A. (2016). Synthetic biology to access and expand nature's chemical diversity. Nature Reviews Microbiology, 14(3), 135–149. 26876034 10.1038/nrmicro.2015.24 PMC5048682

[bib43] Sybesma W. , Blank I., Lee Y.-K. (2017). Sustainable food processing inspired by nature. Trends in Biotechnology, 35(4), 279–281. 28283196 10.1016/j.tibtech.2017.02.001

[bib44] Vink E. T. H. , Rabago K. R., Glassner D. A., Gruber P. R. (2003). Applications of life cycle assessment to NatureWorks^TM^ Polylactide (PLA) production. Polymer Degradation and Stability, 80(3), 403–419.

[bib45] Voigt C. A. (2020). Synthetic biology 2020–2030: Six commercially-available products that are changing our world. Nature Communications, 11(1), 1–6. 10.1038/s41467-020-20122-2 PMC7733420 33311504

[bib46] Wang G. , Haringa C., Noorman H., Chu J., Zhuang Y. (2020). Developing a computational framework to advance bioprocess scale-up. Trends in Biotechnology, 38(8), 846–856. 32493657 10.1016/j.tibtech.2020.01.009

[bib47] Wang H. H. , Isaacs F. J., Carr P. A., Sun Z. Z., Xu G., Forest C. R., Church G. M. (2009). Programming cells by multiplex genome engineering and accelerated evolution. Nature, 460(7257), 894–898. 19633652 10.1038/nature08187 PMC4590770

[bib48] Warner J. R. , Reeder P. J., Karimpour-Fard A., Woodruff L. B. A., Gill R. T. (2010). Rapid profiling of a microbial genome using mixtures of barcoded oligonucleotides. Nature Biotechnology, 28(8), 856–862. 10.1038/nbt.1653 20639866

[bib49] Wetzel S. , Schuffenhauer A., Roggo S., Ertl P., Waldmann H. (2007). Cheminformatic analysis of natural products and their chemical space. CHIMIA International Journal for Chemistry, 61(6), 355–60.

[bib50] Yim H. , Haselbeck R., Niu W., Pujol-Baxley C., Burgard A., Boldt J., Khandurina J., Trawick J. D., Osterhout R. E., Stephen R. (2011). Metabolic engineering of *Escherichia Coli* for direct production of 1, 4-butanediol. Nature Chemical Biology, 7(7), 445–452. 21602812 10.1038/nchembio.580

